# Direct Evidence of Anomalous Interfacial Magnetization in Metamagnetic Pd doped FeRh Thin Films

**DOI:** 10.1038/srep09142

**Published:** 2015-03-16

**Authors:** S. P. Bennett, H. Ambaye, H. Lee, P. LeClair, G. J. Mankey, V. Lauter

**Affiliations:** 1 Quantum Condensed Matter Division, Oak Ridge National Laboratory, Oak Ridge, TN 37830, USA; 2 Research Accelerator Division, Oak Ridge National Laboratory, Oak Ridge, TN 37830, USA; 3 Department of Physics and Astronomy, University of Alabama, Tuscaloosa, AL 35487, USA

## Abstract

Palladium doped iron rhodium is a magnetic material of significant interest for it's close to room temperature magnetostructural phase transition from antiferromagnetic (AF) to ferromagnetic (FM) ordering. Here we report on the peculiarities of the magnetization distribution in thin films of FeRh(Pd) probed by Polarized Neutron Reflectometry. Remarkably, we've found thin interfacial regions with strong magnetization that have unique thermomagnetic properties as compared to the rest of the system. These regions exist at the top and bottom interfaces of the films while the central regions behave similarly to the bulk with a clear AF-FM order transition. Further we explore the impact of an additional Pt interlayer introduced in the middle of the FeRh(Pd) film and reveal that it serves to replicate the strong interfacial magnetization found at the top and bottom interfaces. These results are of great value both in understanding the fundamental physics of such an order transition, and in considering FeRh(Pd) for magnetic media and spintronics applications.

The first order magneto-structural phase transition in thin films of FeRh has been of significant interest for use in next generation heat assisted magnetic recording (HAMR)^1^,^2^,^3^, magnetic refrigeration^1^,^4^,^5^, sensors^6^,^7^ and spintronics^8^,^9^,^10^ applications. Along with a magnetic ordering change from antiferromagnetic (AF) to ferromagnetic (FM), the transition is associated with a 1% expansion of the unit cell^11^,^12^,^13^,^14^, a significantly large magnetoresistance^1^,^15^,^16^,^17^, and an entropy release^1^,^18^,^19^,^20^. Even though the transition is first order, thermomagnetic characterization of FeRh thin films reveals a complex magnetization hysteresis in temperature, giving the critical temperature (T_c_) a range spanning as much as ~100 K in thin films^21^. This hysteresis points towards the nucleation and growth of a network of non-uniform multiphase domains during the phase transformation. Even more remarkably this hysteretic transition has been shown to exist also in high quality epitaxial films^12^,^22^. This has led to a strong drive to understand the underlying physical nature of these unique phase transition phenomena^13^,^22^,^23^,^24^,^25^. Insight into the nucleation of this transition will provide a new understanding of the complex magnetic transitions found in metamagnetic thin films.

With the addition of just 3.0 at.% Pd to the FeRh lattice, the T_c_ for the AF to FM phase transition is lowered close to room temperature^19^,^20^. This decrease in critical temperature is especially valuable for overcoming barriers faced by the development of HAMR magnetic recording^1^,^26^,^27^. As a secondary bonus, lowering the critical temperature allows for detailed study of the magnetostructural phase transition without morphological alteration of the as-grown film due to high temperature characterization^28^. It is therefore of significant interest to study the growth conditions and magnetic characteristics of FeRh(Pd) to determine the distribution of magnetic phases in the system and how they are effected by temperature variation.

A complex hysteretic phase transition, such as that found in FeRh(Pd) films, points towards the presence of a heterogeneous phase domain structure through the whole volume of the film. In this study we reveal the depth dependency of domain formation in FeRh(Pd) single layer and trilayer systems using polarized neutron reflectometry (PNR) at temperatures ranging from 5 K–425 K. Interestingly, we've found thin interfacial regions with strong magnetization that have unique thermomagnetic properties as compared to the rest of the system. These regions exist at the top and bottom interfaces of the films while the central regions behave similarly to the bulk with a clear AF-FM order transition. Further we explore the impact of an additional Pt interlayer and reveal that it serves to replicate in the middle of the FeRh(Pd) film, the magnetization effects found at the top and bottom interfaces.

For this study films were grown on a-plane α-Al_2_O_3_ 1120 sapphire substrates by DC magnetron sputtering from an alloy Fe_46_Rh_48_Pd_6_ target with the target film composition of Fe_46_Rh_48_Pd_6_. Epitaxial growth has widely been achieved on sapphire with metals such as Pt and Nb^29^,^30^,^31^. Both the single and trilayered structures were grown at varying growth temperatures between 400°C–700°C on a combination Rh (~10 nm)/Pt (~10 nm) seed layer, and then capped with Pt (~6 nm) as shown in Figure 1. Characterization of the films was performed using superconducting quantum interference device magnetometry (SQUID) and X-ray diffraction (XRD).

Small changes in the stoichiometry of FeRh_(1−x)_Pd_x_ can have large effects on its magnetic ordering^32^. What makes the system more complex than even FeRh are the three possible magnetic phase orderings that can occur with varying x concentrations from 0–400°C; AF, FM and a FM′ phase. Due to this, and the need for a sufficiently high atomic mobility to obtain good epitaxy and crystallinity, the properties are strongly dependent on the sample temperature during sputter growth. To investigate this growth temperature dependence, 50 nm films of the single layer design were grown at 400°C, 500°C, 600°C and 700°C, then studied by x-ray diffraction (XRD) (Figure 2). Due to the close lattice spacing between Pt, Rh and FeRh(Pd), the layers' Bragg peaks are closely matched in 2θ; making large multimode peaks evident around 40°–42° (in 2θ). Additional pole figure analysis and rocking curves of the FeRh(Pd)/Pt/FeRh(Pd) structure grown on sapphire can be found as supplementary Figure S1 online which confirms epitaxy between layers and with the substrate. To better differentiate between the peaks, an inset in Figure 2 shows a linear intensity scale plot of the peaks between 83°–95° (in 2θ). Three diffraction peaks of FeRh(Pd) are clearly observed in addition to the Pt (222) and Rh (222) peaks for the case of 600°C, 700°C growth temperatures (with positions generated from a Gaussian multi-peak fit). These three diffraction peaks are interpreted as one FCC A1-like L10 (222) and two B2 (220) peaks, one of which has slightly different lattice spacing (labeled by B2′ in the inset)^17^,^33^. The presence of these peaks confirms that a combination of the FCC-like L1_0_, and the CsCl-type B2 phases of FeRh(Pd) grown on Pt 111-oriented seed layer, is energetically favored even with growth temperatures as high as 700°C. A similar coexistence of phases has been seen before for undoped FeRh thin films^7^. They found that when FeRh was grown as a thin film with 43–55% Fe content, a combination of the B2 α′ and the FCC γ was present. Also noticeable upon analysis is a large increase in peak intensities for samples grown at temperatures of 600°C and 700°C. This points to an enhanced epitaxial crystal quality, most probably due to a higher atomic mobility at these elevated temperatures. Increased film quality and a coexistence of phases are also evident from the thermomagnetic properties as measured by SQUID magnetometry in Figure 3. Here it is clear that growth at temperatures of 600°C and 700°C results in films with sharper phase transitions than those at lower temperatures, while also displaying a larger magnetization difference above and below the transition.

We also found that the phase properties of the films are strongly related to the film thickness. Figure 4 shows the thickness dependence investigated by XRD. In the 10 nm thick film the L10 (222) peak is dominant with only a small indication of the B2 (110) peak seen in the 38°–44° 2θ region. This indicates that only a partially ordered B2 structure exists at this film thickness, with the L10 dominating at room temperature. It is not until the thickness reaches 30 nm that the B2′ peak starts to appear. This thickness dependence of the phase concentration also manifests in the thermomagnetic loops taken by SQUID in Figure 5. In these measurements the evolution of the B2 CsCl phase becomes clear and the competition between the phases is evident. At 10 nm thickness there exists primarily the FM phase, which follows a typical increasing magnetization trend with decreasing temperature. A very low amount of the B2 phase is signified by the small hysteretic behavior near the T_c_ region. But as the thickness increases the B2 CsCl phase starts to dominate the magnetic characteristics; shown by a decreasing slope in the low temperature magnetization trend and the hysteretic transition behavior becoming more apparent.

This competition between magnetic orders leads to question where the domains with different phases are distributed and how the domain magnetizations evolve with temperature through the metamagnetic transition. To answer these questions we evolved the depth sensitive method of polarized neutron reflectometry (PNR) on the Magnetism Reflectometer at the Spallation Neutron Source at Oak Ridge National Laboratory. The time of flight instrument has a polarization efficiency of ~98.5%, with a wavelength band of λ ~ 3 Å selected from the wavelength range of 2–8Å of highly polarized neutrons. During the experiment an external magnetic field is applied parallel to the film plane. The reflectivity curves of spin-up (+) and spin-down (−) polarized neutrons are recorded as the function of momentum transfer Q = 4π sin α/λ, where α is the incident angle of the highly collimated neutron beam. The difference in the scattered intensities from the two spin polarization states measured as the function of the momentum transfer Q is determined by the magnetization vector distribution in the sample. From the experimental data we obtain the Nuclear Scattering Length Density (NSLD) and the Magnetic Scattering Length Density (MSLD) depth profiles. MSLD can be directly converted to magnetization using the following relation, MSLD = 2.9 × 10^−7^ (nm^−2^) = 1 (emu/cc). Simultaneous fitting of the data taken at different values of magnetic field and temperatures is used to extract the nuclear and magnetic scattering length density depth profiles. PNR experiments were carried out on two samples, one with the single FeRh(Pd) layer architecture and one with the trilayered design of FeRh(Pd)/Pt/FeRh(Pd), [Figure 1]. The single layer sample was measured at 5 K, 150 K, 172 K, 260 K, 279 K and 300 K with a magnetic field of H = 2 kOe applied in-plane to saturate the sample. The trilayer sample was measured at and around the onset of the transition at temperatures of 5 K, 300 K, 350 K, 425 K and 450 K with a saturation field of H = 1 T. In addition to saturation measurements, the remnance behavior of the magnetization depth profile was measured at 350 K by decreasing the field to 50 Oe.

Python based reflectivity fitting and modeling software GenX^34^ was then used to fit the PNR data [see Figures 6a and 7a]. The structural depth profile NSLD was obtained by fitting the reflectivity data from all temperatures simultaneously whereas the magnetization profile MSLD was varied for different temperatures. From the data analysis we revealed that magnetization within each FeRh(Pd) layer is not uniform. In the model for the trilayered sample each of the two FeRh(Pd) layers was split into 4 sublayers, (labeled FRP_1-8). For the single layer sample 5 sublayers, (labeled FRP_1-5), were necessary to describe the magnetization depth profile.

The structural and magnetization depth profiles obtained from the data analysis are shown in Figures 6b, 6c and 7b. The trilayer and single layer samples show some striking similarities. First is the magnetization at the Pt interfaces. Both films show a large peak in the interfacial magnetization at the bottom interface with the Pt seed layer at 5 K. Also, in the single layer sample especially, a similar peak exists at the top Pt capping layer. In the trilayer sample this peak in magnetization is in addition replicated on top of the Pt interlayer as well. Moreover, unlike the rest of the film, these sublayers have very low remnant magnetizations at 350 K after the field is decreased from 1 T to 50 Oe [Figure 6c]. These interfacial sublayers also share similar decreasing magnetization trends with temperature; void of the clear transition to high magnetization found in the rest of the film. This magnetization temperature trend is reminiscent of FM ordering. Combined with a low remnant magnetization and higher NSLD than for the rest of the film, this could be a signature of a Fe rich region being energetically favored at the interface or diffusion of Pt making FM FeRh(Pt,Pd)^32^,^35^,^36^. Such an enhanced interfacial magnetization was also shown by PNR measurements carried out by R. Fan *et al.* on FeRh films grown on MgO^24^. Since the authors did not notice an increase in the NSLD in the interfacial region, they attributed this interfacial magnetization to anisotropic pressure induced by lattice pinning and not to a likely regional compositional irregularity^23^. This localized strain effect hypothesized in FeRh films could also be present and contributing to the effects seen here.

The strong interfacial FM ordering shown in PNR is confirmed by observations from both SQUID and XRD as a function of film thickness. As the top and bottom interfaces become closer together the interfacial magnetization starts to dominate the films volume and results in a magnetization temperature trend synonymous with ferromagnetism, which is shown in SQUID measurements of the 10 nm thickness (Figure 6). Also, since the XRD data for the 10 nm sample shows the FCC - type L10 phase to be dominant, the interfacial regions could be largely made up of this phase.

Interesting too is the clear differences in T_c_′s of the sublayers away from the interfaces in the trilayer sample. This is most likely due to an intermixing of different magnetic phases in the plane of the specimen^37^. The work recently carried out by C. J. Kinane *et al.* shows how the in-plane surface domains evolve through the temperature transition^22^. In their work x-ray photoemission electron microscopy (XPEEM) was used to show that the surface layer has a mixture of AF and FM domains that have different pattern symmetries depending on the competing magnetic orders through the transition temperature. Here we show that this lateral domain evolution has a 3-dimentional nature with the depth dependence, and exists for each separate sublayer shown in PNR. Further research combining PNR and XPEEM could lead to a more complete picture on how these domains are behaving at the different sublayers and how their evolution affects the sublayer magnetization characteristics in metamagnetic thin films.

In conclusion, by controlling growth temperature and layer thicknesses, FeRh(Pd) single layer and trilayer films were grown with T_c_′s in the range of 200–300 K. XRD and SQUID magnetometry point towards a competition between a FM and FM′ phase dominating the thermomagnetic characteristics of the films. The findings also show that the films magnetization and phase concentration depend strongly on film thickness. The structural and magnetization depth profiles were then studied in the vicinity of the phase transition using Polarized Neutron Reflectometry (PNR) at temperatures ranging from 5 K to 425 K for the tri-layer film, and 5 K to 300 K for the single layer. PNR reveals regions at the FeRh(Pd)/Pt interfaces in both samples that have clear FM ordering characteristics as a function of temperature, while regions away from the interfaces show a clear metamagnetic phase transition in the T_c_ region. Furthermore, this interfacial FM region is replicated at the interface of a Pt interlayer in a trilayer film. Our findings answer long standing questions about the hysteretic behavior and growth condition dependency of the magnetic phase transition in FeRh(Pd) thin films and take us a step closer to fully understanding metamagnetic transitions in low dimensional epitaxial systems.

## Author Contributions

**Figure 1:**
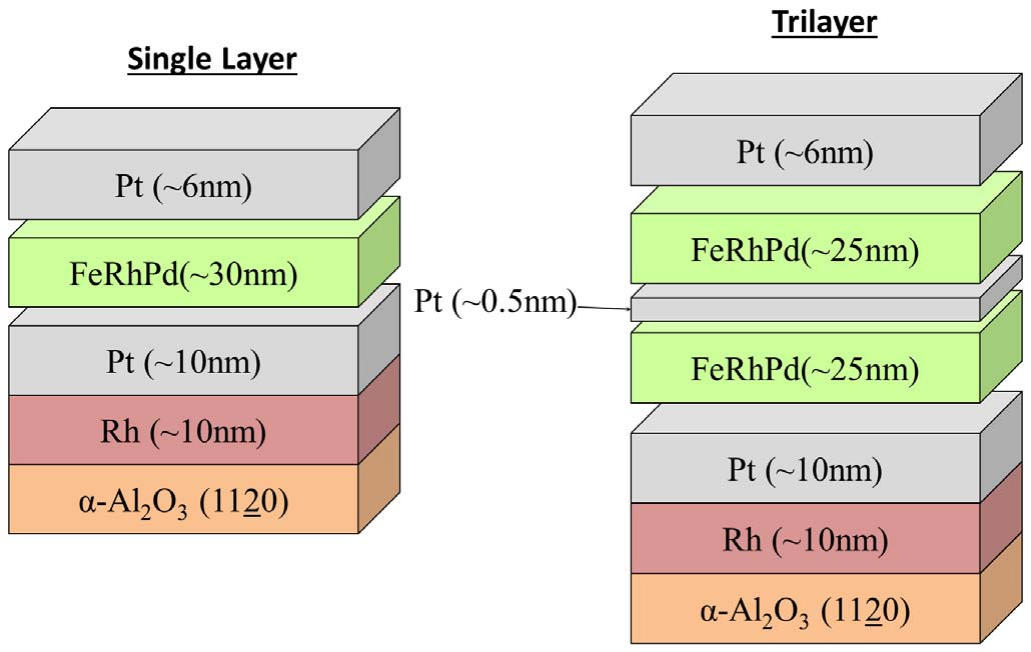
Sample structure for the FeRh(Pd) single layer and trilayer FeRh(Pd)/Pt/FeRh(Pd) designs. Nominal values for layer thicknesses are labeled however variations in deposition rate led to the measured thicknesses shown in Figures 6 and 7.

**Figure 2:**
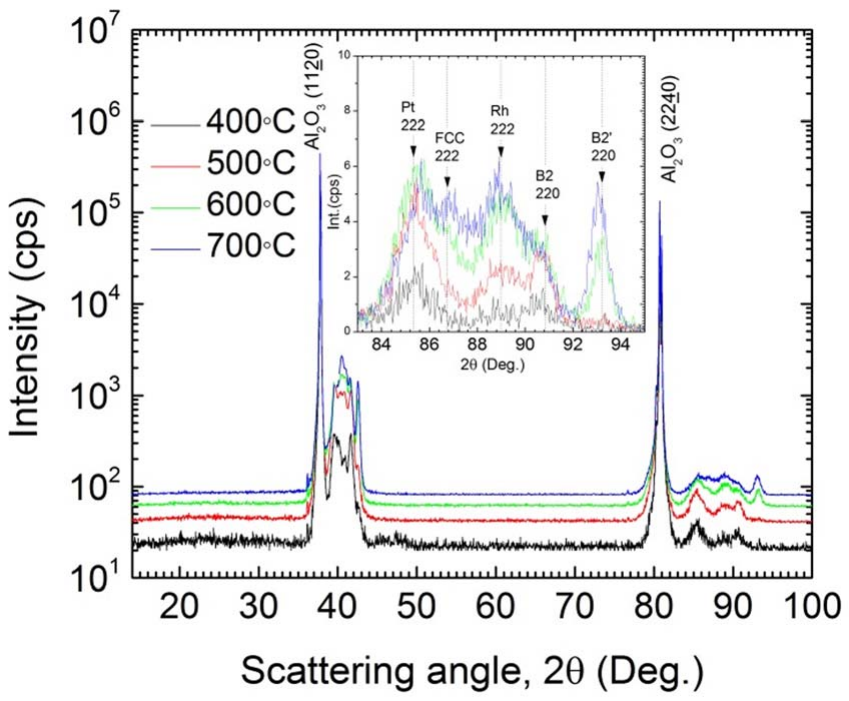
X-ray diffraction data for single layer FeRh(Pd) films grown at 400°C, 500°C, 600°C and 700°C. Each temperature was shifted in intensity for visual clarity. The inset shows peaks from the Pt and Rh buffer layers, (Pt 222, Rh 222), as well as peaks from FeRh(Pd), (FCC 222, B2 220, B2′ 220).

**Figure 3:**
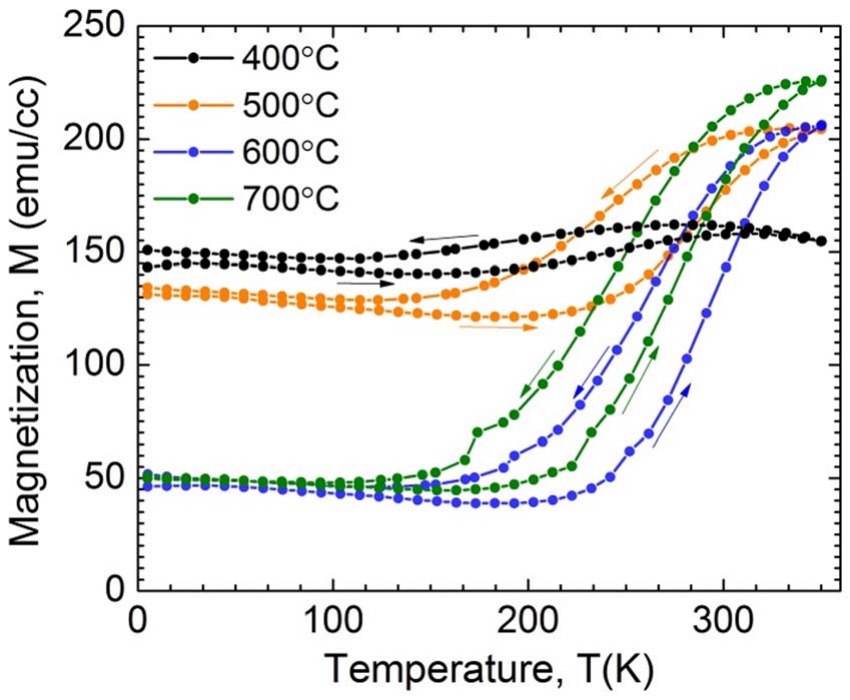
Saturation magnetization versus temperature measurements from 5–360 K taken with a SQUID magnetometer with 1Tesla applied field on 50 nm thick single layer samples grown with varying growth temperatures. Arrows indicate temperature sweep direction.

**Figure 4:**
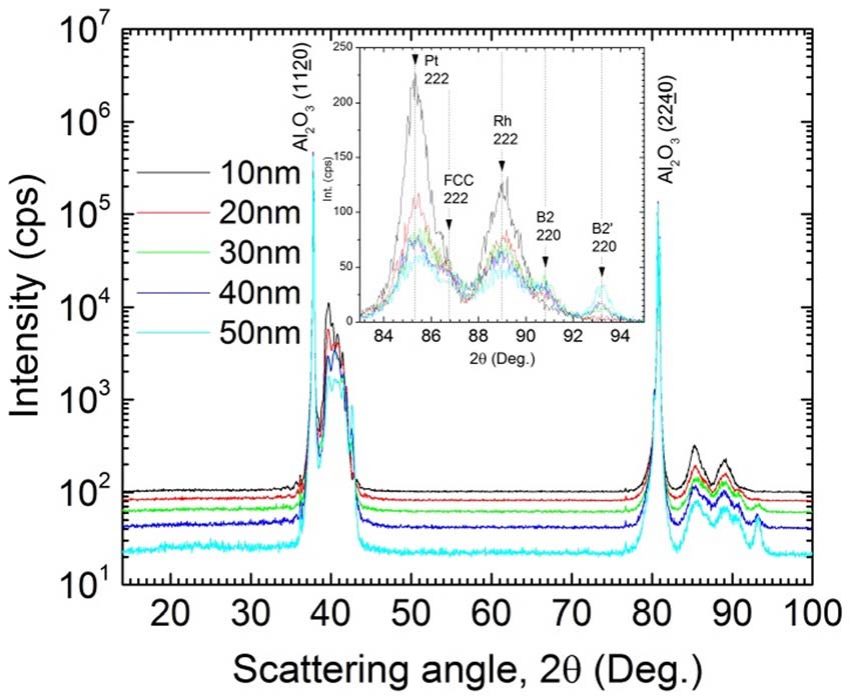
X-ray diffraction data for FeRh(Pd) single layer films grown at 600°C with varying layer thickness. The inset shows peaks from the Pt and Rh buffer layers, (Pt 222, Rh 222), as well as peaks from FeRh(Pd), (FCC 222, B2 220, B2′ 220). Decreasing Pt/Rh layer peak intensities indicate an increase in interdiffusion between the layers likely due to the increased heating time for the thicker films.

G.J.M., H.L. and P.L. grew the samples and performed SQUID magnetometer measurements. H.L., H.A. and V.L. conducted polarized neutron reflectometry experiments. S.B. compiled and fit neutron reflectometry data. S.B. prepared the manuscript while H.L. contributed figures 1–4. All authors contributed to discussion and revision of the article.

## Supplementary Material

Supplementary Information Supplementary Figure S1 and Figure S2

## Figures and Tables

**Figure 5:**
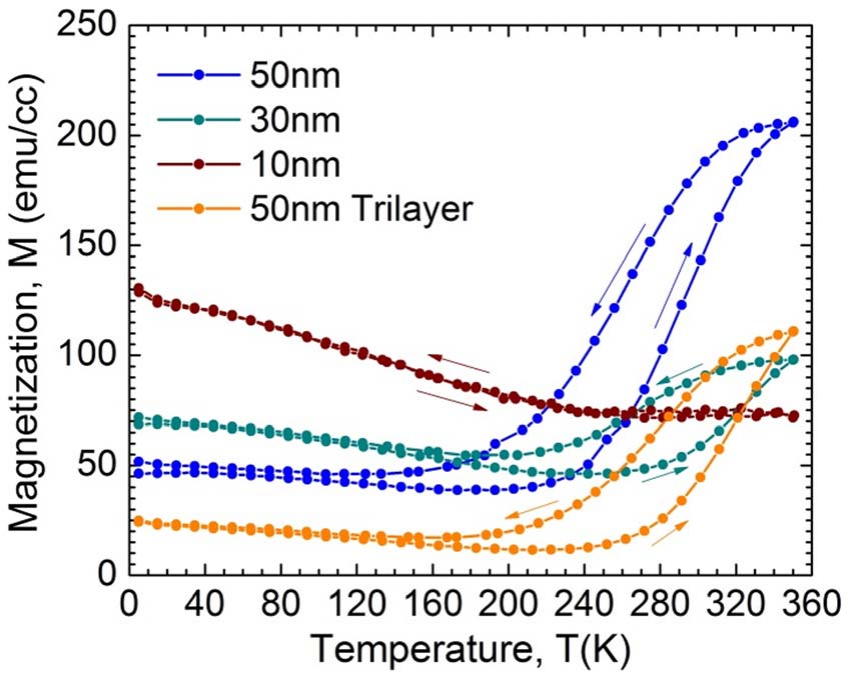
Saturation magnetization curves as a function of temperature for FeRh(Pd) single layer films with 1 Tesla applied field grown at 600°C with varying thicknesses, (50 nm, 30 nm and 10 nm). Arrows indicate temperature sweep direction.

**Figure 6:**
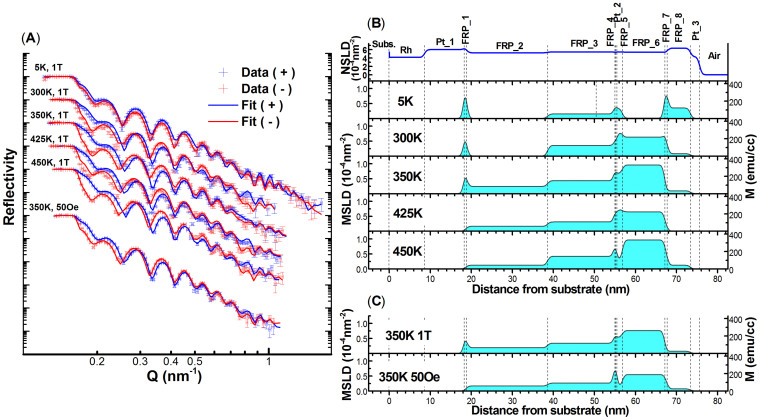
(A) PNR reflectivity data for the trilayer sample taken at 5 different temperatures as a function of momentum transfer Q. The data points are designated with error bars and the fitted models are shown as solid lines, where blue (+) corresponds to neutrons with spin parallel to the direction of the external field, and red (−) corresponds to neutrons with spins anti-parallel. In addition the sample was measured at 50 Oe for the 350 K after being saturated in 1 T. (B) The structural (NSLD) and magnetic (MSLD) scattering length density depth profiles obtained from the fit to the data for each temperature are shown as functions of the distance from the substrate surface, (with corresponding vertical axis on the left side). Corresponding values of magnetization are presented in the right side vertical axis. (C) A comparison between the magnetization depth profiles taken at 350 K at saturation (M_s_) at 1 T and remnance (M_r_) at 50 Oe.

**Figure 7:**
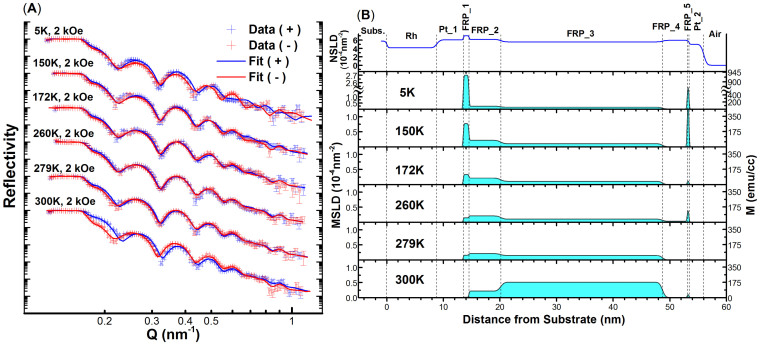
(A) PNR reflectivity data for the single layer sample taken at 6 different temperatures as a function of momentum transfer Q. The data points are designated with error bars and the fitted models are shown as solid lines, where blue (+) corresponds to neutrons with spin parallel to the direction of the external field, and red (−) corresponds to neutrons with spins anti-parallel. (B) The structural (NSLD) and magnetic (MSLD) scattering length density depth profiles obtained from the fit to the data for each temperature are shown as functions of the distance from the substrate surface, (with corresponding vertical axis on the left side). Corresponding values of magnetization are presented in the right vertical axis.
