# Supplementary material for: Direct Evidence of Anomalous Interfacial Magnetization in Metamagnetic Pd doped FeRh Thin Films
Source: Sci Rep. 2015 Mar 16;5:9142. doi: 10.1038/srep09142 (PMC4360473; doi:10.1038/srep09142)
Supplement: Supplementary Information — Supplementary Figure S1 and Figure S2 [file srep09142-s1.pdf]

Direct Evidence of Anomalous Interfacial Magnetization in Metamagnetic Pd doped FeRh Thin Films

S. P. Bennett[1], H. Ambaye[2], H. Lee[3], P. LeClair[3], G. J. Mankey[3], V. Lauter[1]

Supplementary Data:

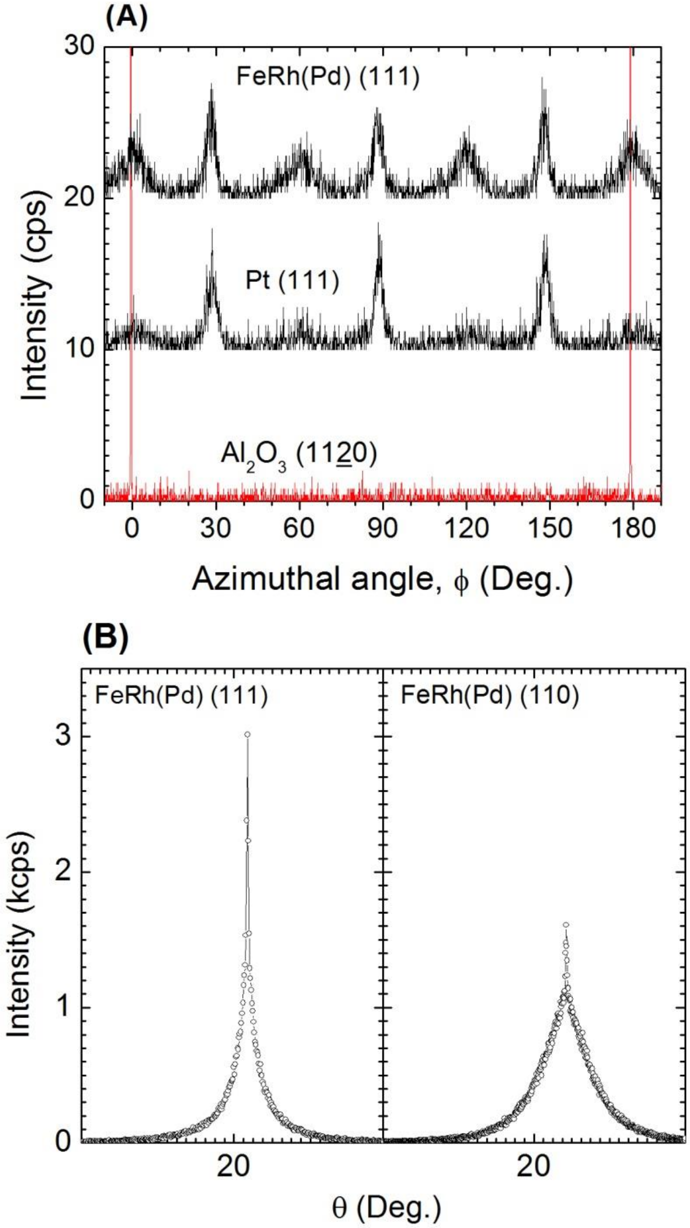

Supplementary Figure S1: (A) Pole figures of trilayered FeRh(Pd) (25nm)/Pt (0.5nm)/FeRh(Pd) (25nm) structure grown on a sapphire substrate. The α-Al2O3 (1120) and Pt (111) layers were measured at the tilted angle of 60°, and the FeRhPd (111) at the tilted angle of 70.5°. Data for Pt and FeRh(Pd) were shifted by 10 counts per second for comparison. (B) Rocking curves measured for $L1_0$ FeRh(Pd) (111) and B2 FeRh(Pd) (110).

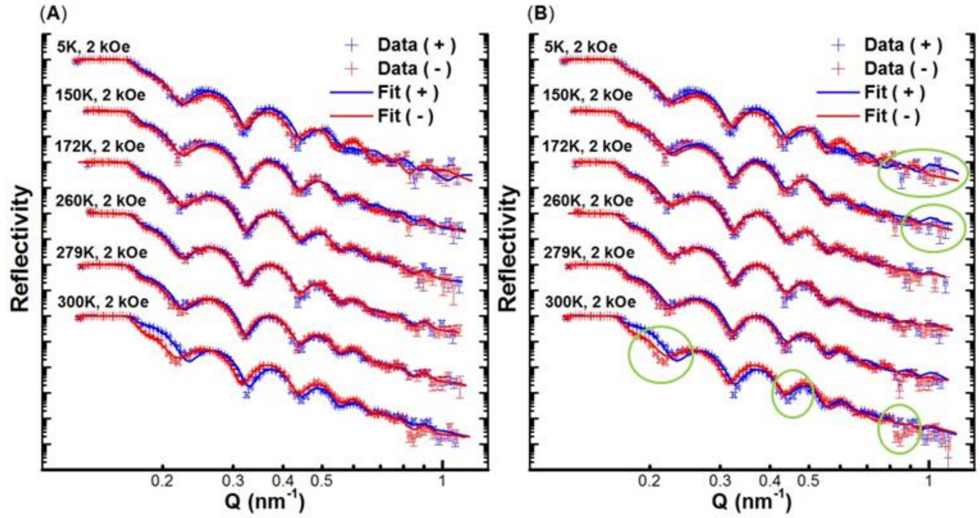

Supplementary Figure S2: (A) PNR reflectivity data and fit of single layer film from Figure 7 in text (shown for comparison). (B) PNR reflectivity data and fit of a single layer film with a fixed NSLD of 5.5 (x10^{-4}nm^{-2}) for sublayer FRP_2. This shows how the PNR method is sensitive to the NSLD of this particular sublayer. Green circles indicate specific areas where the fit suffered from fixing this value to a lower number.
